# The Effect of Fatigue Test on the Mechanical Properties of the Cellular Polyurethane Mats Used in Tram and Railway Tracks

**DOI:** 10.3390/ma14154118

**Published:** 2021-07-23

**Authors:** Juliusz Sołkowski, Jarosław Górszczyk, Konrad Malicki, Dariusz Kudła

**Affiliations:** Faculty of Civil Engineering, Cracow University of Technology, 31-155 Cracow, Poland; jsolkow@pk.edu.pl (J.S.); kmalicki@pk.edu.pl (K.M.); dkudla@pk.edu.pl (D.K.)

**Keywords:** tram-railway vibration, tram and railway tracks, cellular polyurethane (PUR), vibro-acoustic isolators, dynamic bedding modulus, under-ballast mats, high cycle fatigue (HCF)

## Abstract

The use of modern synthetic materials is an important element in the development of railway tracks. Their use is a response to the growing requirements regarding the durability of structures and environmental protection against traffic noise and vibrations. In this paper, the results of the laboratory tests of selected mechanical properties of cellular polyurethane (PUR) mats which are applied in tram and railway tracks are presented in this study. The aim of the research was to determine the effect of fatigue loading on the mechanical performance of polyurethane mats. A series of samples made of two types of materials with different pore structures were tested. Static and fatigue laboratory tests were carried out on a specially prepared test stand. The values of selected mechanical parameters (the vertical static bedding modulus, the vertical dynamic bedding modulus, and the loss factor) were evaluated. The results of laboratory tests and analyses showed a significant influence of high-cycle fatigue loading on the values of mechanical parameters of the tested mats, which were quantified as a result of the study. For both types of materials, the phenomenon of cyclic hardening was observed. Additionally, for one of the materials, an undesired dynamic creep phenomenon was observed. It was also shown that the pore structure of polyurethane influences the mechanical performance of the mats. Therefore, the findings of the research may have practical significance for the quality evaluation of such materials, especially in the context of their durability and mechanical stability under real loading conditions.

## 1. Introduction

The structures of tram and railway tracks are constantly refined through the use of modern building materials and technologies. This process is often associated with the need to meet increasing requirements regarding the durability and recycling of structures, as well as the protection of people and the environment against traffic noise and vibrations. The implementation of the research and analyses aimed at quantifying the mechanical properties of modern building materials is, thus, a key issue from scientific, engineering, social, and environmental points of view [1,2,3,4,5,6]. Products made of synthetic materials constitute a wide group of modern materials used more and more often in transport infrastructure. For example, geosynthetics are widely used in the lower layers of railway and road structures [7,8,9,10,11,12]. They can perform well in several different functions. Reinforcing and separation-filtration layers are the most common. Synthetic materials can also be used in the upper layers of the structure. Various types of mats and pads are used in tram and railway tracks [2,13,14,15]. One of these types of mats is a polyurethane mat. According to Sol-Sanchez et al. [15], the installation of elastic elements in the railway track has now become the most effective means to vary track stiffness, as well as to reduce noise emission and vibrations caused by trains.

The parameters and procedures used in testing the mechanical properties of mats for vibration isolation of railway tracks are described in the German standards [16,17,18]. They were also discussed by Kraśkiewicz et al. and Sol-Sanchez et al. [2,15]. The most important parameters include static and dynamic vertical and horizontal stiffness, static and dynamic bedding modulus, and loss factor, defined as the ratio of energy dissipated to energy expended. Bedding modulus and stiffness determine the effectiveness of dumping vibration transmission to the environment [2,13]. Mats with a very low value of static bedding modulus or stiffness cause greater vertical deflection of rail and railway track. This causes greater stresses and reduces the fatigue life of the railway track. The influence of the fatigue process on the mechanical parameters of materials and components used in transport infrastructure was discussed e.g., in [19,20,21,22]. Fatigue processes usually deteriorate the tested mechanical parameters.

Diego et al. [3] presented numerical and experimental tests of the mechanical parameters of elastomer mats used for vibration isolation in railway tracks. The mats were made of recycled materials. Tests were carried out for determining moduli, the behavior under fatigue conditions and the ability of the material to resist mechanical actions in the presence of frost and aging factors. The static and dynamic moduli obtained with ballast plates were lower than those obtained using steel plates. An increase in stiffness with increasing load frequency and a decrease in stiffness with increasing temperature were observed. In summary, this material is a suitable solution to be used as an anti-vibration system for railway applications, its mechanical properties meet the requirements for such a type of components. The average dynamic modulus and loss factor of the tested mat using a ballast plate was equal to 0.0267 N/mm^3^ and 0.423 and using steel plate 0.0373 N/mm^3^ and 0.427 for a loading frequency of 5 Hz [3]. According to Hanson and Singleton [23], the model dynamic stiffness of ballast mats on passenger railroads may be taken as equal to 0.014 N/mm^3^.

The values of mechanical parameters determined in laboratory tests are used in theoretical and numerical investigation as well as in experimental tests of the entire structure of the railway track. These analyses were conducted by, for example, Czyczuła et al. [24]. Analytical evaluation of track response due to a moving load was considered. The subject of experimental validation of a wavelet-based solution for dynamic response of railway track subjected to a moving train was taken up by Kozioł [25]. Analyses of the transition effect in railway tracks with different types of support or with a sudden change of foundation stiffness are also carried out. Such issues were considered by, for example e.g., Dimitrovowa and Varandas [26] and Sołkowski [14]. Numerical simulations and theoretical models are used in the analysis. Parameters concerning damping and stiffness of the track components are used there.

This paper presents the results of laboratory tests of selected mechanical parameters of polyurethane mats used in tram and railway tracks. A series of samples made of materials with different properties were tested. A specially prepared test stand was used, based on a servo-hydraulic testing system. Procedures referring to the German standards [16,17,18] were applied. The static and dynamic bedding modulus and the loss factor of the tested materials were determined. The influence of fatigue test on the change of these parameters was shown and quantified. Cyclic loads were used to simulate service conditions. The values of the mechanical parameters of modern mats made of cellular polyurethane subjected to the fatigue process quantified in this study have scientific and practical significance. The results of these studies can be used in further theoretical analysis, as well as in the optimization of material durability by manufacturers.

## 2. Materials and Methods

### 2.1. Aim of the Tests, Characteristics of Tested Materials, and Sample Preparation

**Figure 1:**
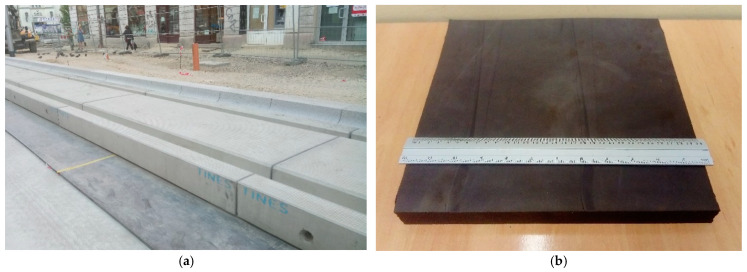
(**a**) Vibroinsulating mat built into the street tram track; (**b**) sample prepared for testing (300 × 300 × 25 mm³).

The aim of the research was to determine the effect of high-cyclic loads (high cycle fatigue test—HCF) on the change in the value of selected mechanical parameters of vibroinsulating mats used in tram and railway tracks. These types of mats are used, for example, in floating slab tracks. The polyurethane mat placed underneath the concrete slab of the tram track is shown as an example in Figure 1.

Two types of mats made of cellular polyurethane (PUR) with different pore structure and mechanical parameters were tested. In this article, the mats are marked with letters A and B. The technical data of Mat A and Mat B are given in [27]. The static range of use for Mat A can be up to 0.015 N/mm^2^ and for Mat B up to 0.020 N/mm^2^. Square samples with side dimensions of 300 mm were prepared for the tests by cutting them out of mats. The thickness of the samples was 25 mm. Six samples (300 × 300 × 25 mm^3^) were made for each type of mat. The view of the sample prepared for testing is shown in Figure 1.

### 2.2. Test Methods and Parameters

Static and dynamic compression tests, as well as fatigue tests, were performed on a laboratory stand that met the standard requirements [18]. The tests were performed on 6 samples prepared from each of the tested mats, and the final results were averaged for the tested series.

The load characteristic was adopted (equivalent to the stress) reflecting the operating conditions of mats in the tram track. The load range has been established on the basis of the manufacturer’s recommendations in the technical approval [27]. The parameters of static tests are presented in Table 1. In the dynamic and fatigue tests, the harmonic load, and the method of controlled force (stress) were used. Cyclic loads with the characteristics presented in Table 2 were applied. The number of cyclic loads was assumed at the level of 3 × 10^6^ cycles. This corresponds to an estimated 6 years of use of the tram track with 10-axle trams passing every 10 min around the clock. The tests were carried out at a temperature equal to 23 ± 1 °C (room temperature).

The laboratory test program included the following steps and parameters for each sample:

- determination of the initial vertical static bedding modulus C_stat_1_ according to Equation (1) [17] and Table 1;
- determination of the initial vertical dynamic bedding modulus C_dyn_1_ according to Equation (1) [17] and Table 2;
- determination of the initial loss factor η according to Equations (2) and (3) [17] and Table 2;
- fatigue test—3 million cyclic loads with the characteristics specified in Table 2
- redetermination of the vertical static bedding modulus C_stat_2_ (for samples after the fatigue test);
- e-determination of the vertical dynamic bedding modulus C_dyn_2_ (for samples after the fatigue test);
- re-determination of the loss factor η (for samples after the fatigue test):
  (1) $$C_{\mathrm{stat}/\mathrm{dyn}}=\frac{\Delta F}{\Delta \delta }\;(N/{\mathrm{mm}}^{3})$$
  where ΔF (N/mm^2^) is the load change, Δδ (mm) is the change in mat thickness due to the change in load ΔF:
  η = tan ζ(2)
  ζ = Δt·f·2π(3)
  where η is the loss factor, ζ is the loss angle determined as the angular phase shift Δt (s) between the applied harmonic force and the resulting deformation, f (Hz) is the load frequency.

### 2.3. Laboratory Set-Up

**Figure 2:**
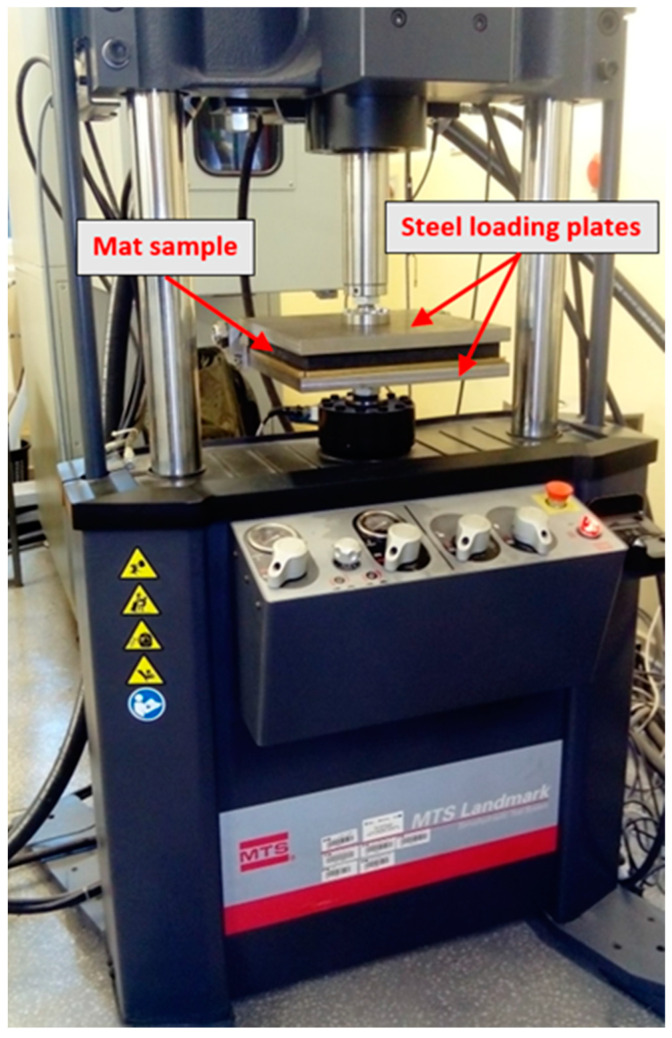
Laboratory stand with the mat sample.

The laboratory stand is based on the servo-hydraulic testing system. This system can be used to perform static and dynamic/fatigue testing of samples of various materials and structural components. The accuracy class of force transducers equals 0.5 in a range of the force value from 1% to 100%. The laboratory stand, with the sample prepared for the test, is shown in Figure 2.

The direct application of the compressive load was carried out on the entire upper and lower surface of the samples through steel plates ideally suited to the dimensions of the samples (Figure 2).

## 3. Results and Discussion

**Figure 3:**
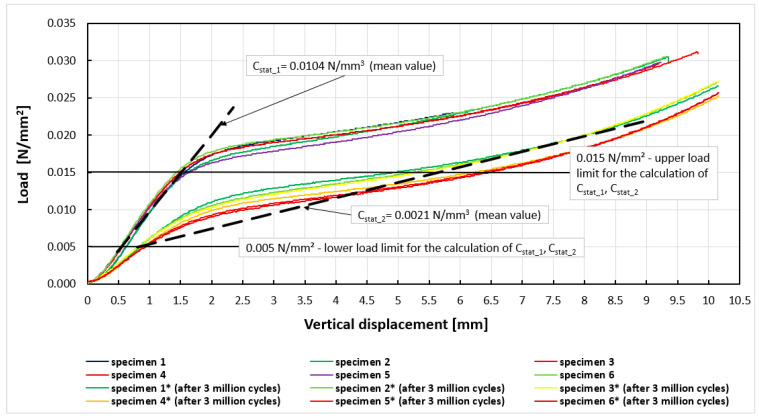
Experimental curves (load vs. displacement) for determining C_stat_1_, C_stat_2_ moduli for Mat A.

**Figure 4:**
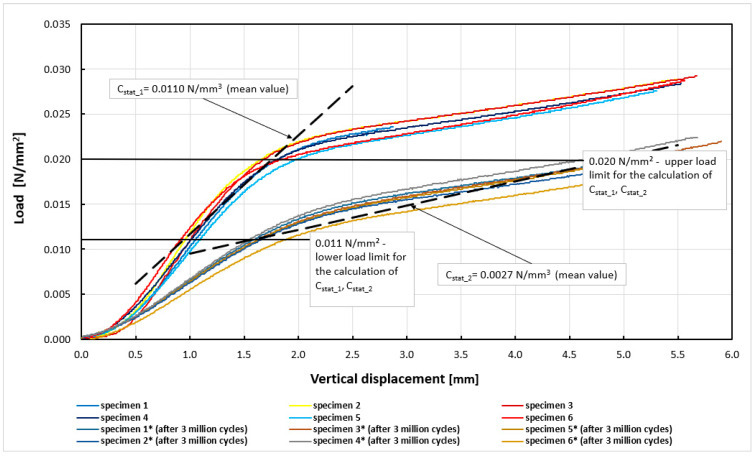
Experimental curves (load vs. displacement) for determining C_stat_1_, C_stat_2_ moduli for Mat B.

The first stage of the research involved determination of experimental curves, static load vs. displacement for both types of mats. Next, the lower and upper load limits for calculating the moduli were marked. The curves obtained and lines used to determine the mean values of the static moduli are shown in Figure 3 and Figure 4. Table 3 and Table 4 show a comparison of the results of the static module tests before and after the fatigue tests.

The static bedding modulus C_stat_ for Mat A before the fatigue test was equal to 0.0104 ± 0.0007 N/mm^3^ (mean ± standard deviation). After the fatigue test, the bedding modulus was 0.0021 ± 0.0003 N/mm^3^. The modulus C_stat_ for Mat B before the fatigue test was equal to 0.0110 ± 0.0016 N/mm^3^, and after the fatigue test, the value of the static bedding modulus was 0.0027 ± 0.0003 N/mm^3^. The static bedding modulus C_stat_ of Mat A after the fatigue loading decreased by 80% compared to the unloaded mat. For Mat B, this decrease was approximately 76%. These are quite high values. However, it should be noted that the results of both mats could have been influenced by the assumption made during the static loading tests. The mats were loaded from a “zero” force to a force, such as to obtain the maximum vertical displacement according to the technical specification [27]. This method of loading does not correspond to the real loading conditions in the railway track, which is a structure mainly used under dynamic loading. In the static tests, higher values of the coefficients of variation were also obtained than in the dynamic tests, which were performed at the average stress value (the constant stress value). This static study has, thus, a rather comparative character.

**Figure 5:**
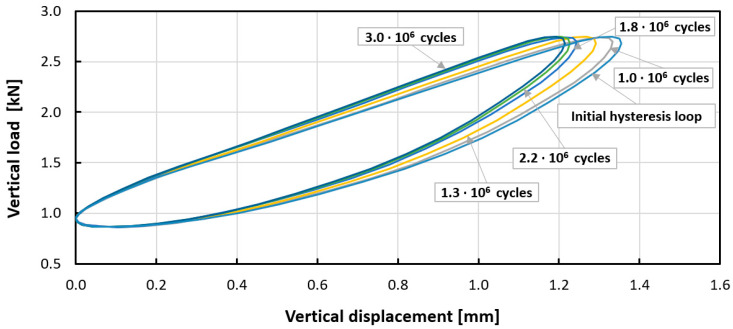
Experimental curves (specimen 3) showing the effect of the number of load cycles on the dynamic mechanical response (C_dyn_), cyclic hardening of Mat A.

**Figure 6:**
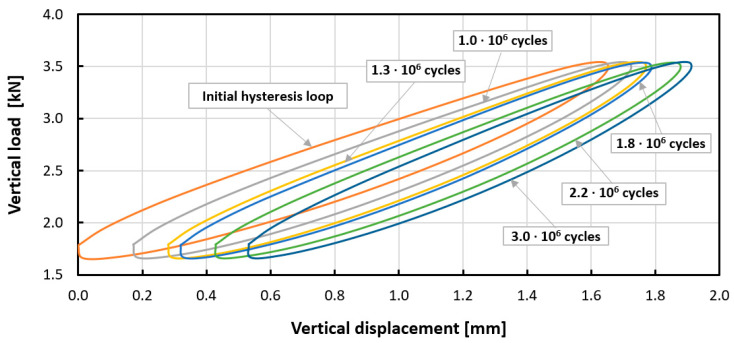
Experimental curves (specimen 2) showing the effect of the number of load cycles on the dynamic mechanical response (C_dyn_), cyclic hardening and dynamic creep of Mat B.

**Figure 7:**
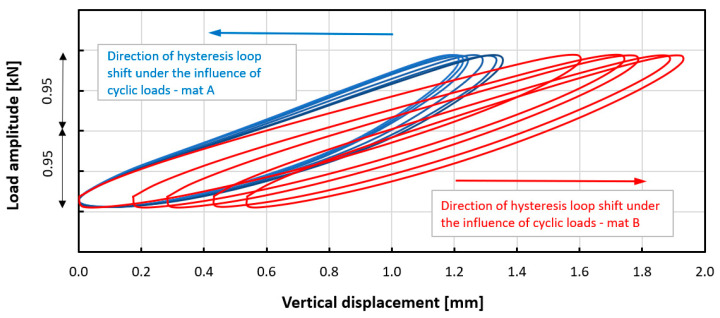
Comparison of experimental curves showing the effect of the number of load cycles on the dynamic response of both types of mats, dynamic creep phenomenon can be observed for Mat B (hysteresis loops transformed to mean load value = 0).

In the next stage, dynamic tests were carried out. Experiment curves showing the effect of the number of load cycles on the dynamic mechanical response of tested mats are shown in Figure 5 and Figure 6. For both mats, the phenomenon of cyclic hardening was observed, which changed the value of the dynamic modulus with the progress of the fatigue process. The mechanism of fatigue hardening is based on an increase in dynamic stiffness/dynamic modulus with increasing load cycles (Table 5 and Table 6). For Mat B during fatigue loading, the phenomenon of dynamic creep (growth of permanent deformation under the influence of cyclic loads) was additionally observed. This is not a desired phenomenon. This can be seen in Figure 6 and Figure 7. This phenomenon is caused by the different pore structure of the tested materials. Based on the results obtained, the dynamic bedding modulus was determined as a function of the number of applied load cycles. The results for Mat A and Mat B are summarized in Table 5 and Table 6.

The initial dynamic bedding modulus C_dyn_ is defined as the quotient of the load amplitude to the mat displacement amplitude in the initial phase of the cyclic load (in the 200th cycle) and was equal to 0.0154 ± 0.0005 N/mm^3^ (mean ± standard deviation) for Mat A and 0.0128 ± 0.0005 N/mm^3^ for Mat B. After the fatigue test (after 3 million load cycles), the dynamic stiffness modulus C_dyn_ was equal to 0.0178 ± 0.0005 N/mm^3^ for Mat A and 0.0147 ± 0.0004 N/mm^3^ for Mat B, that is it increased by 15% for Mat A and 14% for Mat B in relation to the modulus value at the beginning of the test. This proves the significant mechanical stability of both mats and the efficiency throughout the entire period of use. The dynamic bedding modulus and the loss factor are better parameters to describe the real load conditions and the efficiency of mats in a railway track than C_stat._

**Figure 8:**
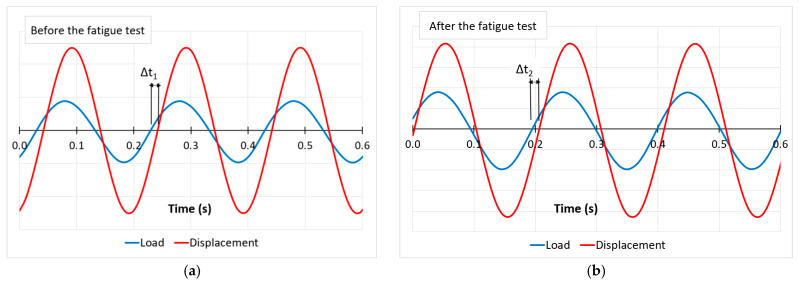
Example of load and displacement variability of Mat B (specimen 2) in 0.6 sec (cycles transformed to mean value = 0). The phase shift (delay) of the displacement is: (**a**) Δt_1_ = 0.01311 s (before the fatigue test) and (**b**) Δt_2_ = 0.01291 s (after the fatigue test).

In dynamic tests, the variability of force and displacement was recorded. This record was used to determine the phase shift of the displacement Δt. On this basis, the value of the loss factor η was determined according to Equations (2) and (3). An example of determining the phase shift for sample 2 prepared from Mat B is shown in Figure 8. The average values of the loss factor obtained in this way for Mat A and Mat B are shown in Table 7 and Table 8.

The change in the value of the loss factor η under the fatigue load was equal to 1.2% for Mat A, a decrease from 0.4979 to 0.4917, and 1.7% for Mat B, a decrease from 0.4370 to 0.4294. The value of the loss factor is one of the measures of attenuation of the energy emitted from a vibrating object (for example e.g., rails or other component of the railway track) to its surroundings. It determines the ratio of the dissipated energy value to the load energy value. The higher the value of the loss factor η, the better the vibroacoustic insulator. The results obtained are similar to those presented in the literature [3] and confirm good mechanical stability of both mats.

## 4. Conclusions

The effect of fatigue loading on the mechanical properties of the selected cellular polyurethane mats has been tested and quantified. The results of the research formulate the following conclusions:

- The static bedding modulus C_stat_ of Mat A after the fatigue test decreased by about 80% compared to the unloaded mat. For Mat B, this decrease was approximately 76%. Such results could have been caused by adopted static test conditions;
- For Mat A and Mat B, the phenomenon of cyclic hardening was observed; additionally, for Mat B, dynamic creep was observed, which is not a desired phenomenon;
- An important result of the research carried out here is the determination of the significant difference in the behavior of the compared mats under high-cyclic loads. It was shown that the pore structure of polyurethane influences the mechanical performance of the mats. The phenomena observed indicate the possibility of further optimization of mat parameters;
- The dynamic bedding modulus and the loss factor respond better than the static parameters to the real loading conditions of the mats in the tram and railway tracks;
- The value of the dynamic bedding modulus C_dyn_ increased under the fatigue loading by approximately 15% for Mat A and 14% for Mat B. The change in the value of the loss factor η under the fatigue test was less than 2% for both mats. This proves the good mechanical stability of the materials tested according to [16,17,18] standards. Mechanical stability should be understood as the change of mechanical parameters after fatigue loading. The results obtained regarding the dynamic bedding modulus are similar to those presented in the literature [3,23];
- The findings of the research may have a practical significance for the quality evaluation of such materials, especially in the context of their durability and mechanical stability under real loading conditions.

## Figures and Tables

**Table 1:** Static load characteristics for two types of mats.

Static Test Parameters	Mat A	Mat B
lower load limit for the C_stat_1_, C_stat_2_	0.005 N/mm^2^ (0.45 kN)	0.011 N/mm^2^ (0.99 kN)
upper load limit for the C_stat_1_, C_stat_2_	0.015 N/mm^2^ (1.35 kN)	0.020 N/mm^2^ (1.80 kN)
displacement loading rate	0.5 mm/min.	0.5 mm/min.

**Table 2:** Cyclic load (fatigue loading) characteristics for two types of mats.

Fatigue Test Parameters	Mat A	Mat B
Mean value	0.02 N/mm^2^ (1.80 kN)	0.03 N/mm^2^ (2.55 kN)
Amplitude	0.01 N/mm^2^ (0.95 kN)	0.01 N/mm^2^ (0.95 kN)
Amplitude ratio	0.50	0.35
Load frequency	5.0 Hz	5.0 Hz
Total number of cycles	3 × 10^6^	3 × 10^6^

**Table 3:** Experimental results of the static bedding modulus C_stat_ for Mat A.

	C_stat___1_ (Before the Fatigue Test)	C_stat___2_ (After the Fatigue Test)	C_stat_2_/C_stat_1_
mean value (N/mm^3^)	0.0104	0.0021	0.20
standard deviation (N/mm^3^)	0.0007	0.0003	0.40
coefficient of variation (%)	6.5	12.8	1.99

**Table 4:** Experimental results of the static bedding modulus C_stat_ for Mat B.

	C_stat___1_ (Before the Fatigue Test)	C_stat___2_ (After the Fatigue Test)	C_stat_2_/C_stat_1_
mean value (N/mm^3^)	0.0110	0.0027	0.24
standard deviation (N/mm^3^)	0.0016	0.0003	0.19
coefficient of variation (%)	14.2	11.1	0.78

**Table 5:** Experimental results of the dynamic bedding modulus C_dyn_ as a function of the number of load cycles for Mat A.

		Number of Cycles					
		200 (Initial)	1.0 × 10^6^	1.3 × 10^6^	1.8 × 10^6^	2.2 × 10^6^	3.0 × 10^6^
C_dyn_ (N/mm^3^)	specimen 1	0.0164	0.0165	0.0169	0.0173	0.0178	0.0181
	specimen 2	0.0156	0.0158	0.0162	0.0166	0.0169	0.0171
	specimen 3	0.0155	0.0157	0.0162	0.0168	0.0171	0.0172
	specimen 4	0.0150	0.0152	0.0158	0.0162	0.0173	0.0178
	specimen 5	0.0149	0.0162	0.0165	0.0169	0.0181	0.0185
	specimen 6	0.0153	0.0159	0.0164	0.0172	0.0176	0.0179
mean value C_dyn_ (N/mm^3^)		0.0154	0.0159	0.0163	0.0168	0.0175	0.0178
standard deviation C_dyn_ (N/mm^3^)		0.0005	0.0004	0.0004	0.0004	0.0005	0.0005
coefficient of variation (%)		3.5	2.7	2.2	2.4	2.6	3.0
ΔC_dyn_ (%)—Mean value		0	+3	+6	+9	+13	+15
(change in mean C_dyn_ from the value calculated over the 200th cycle)							

**Table 6:** Experimental results of the dynamic bedding modulus C_dyn_ as a function of the number of load cycles for Mat B.

		Number of Cycles					
		200 (Initial)	1.0 × 10^6^	1.3 × 10^6^	1.8 × 10^6^	2.2 × 10^6^	3.0 × 10^6^
C_dyn_ (N/mm^3^)	specimen 1	0.0129	0.0135	0.0137	0.0139	0.0140	0.0147
	specimen 2	0.0127	0.0135	0.0140	0.0141	0.0144	0.0151
	specimen 3	0.0131	0.0133	0.0139	0.0140	0.0143	0.0150
	specimen 4	0.0123	0.0129	0.0134	0.0136	0.0139	0.0141
	specimen 5	0.0137	0.0138	0.0142	0.0144	0.0147	0.0150
	specimen 6	0.0123	0.0127	0.0129	0.0131	0.0139	0.0142
mean value C_dyn_ (N/mm^3^)		0.0128	0.0133	0.0137	0.0139	0.0142	0.0147
standard deviation C_dyn_ (N/mm^3^)		0.0005	0.0004	0.0005	0.0005	0.0003	0.0004
coefficient of variation (%)		4.1%	3.1%	3.4%	3.3%	2.2%	3.0%
ΔC_dyn_ (%)—Mean value		0	+3	+7	+8	+11	+14
(change in mean C_dyn_ from the value calculated over the 200th cycle)							

**Table 7:** Experimental results of the loss factor η for Mat A.

Loss Factor	η_1_ (Before The Fatigue Test)	η_2_ (After The Fatigue Test)	η_2_/η_1_
mean value	0.4979	0.4917	98.8%
standard deviation	0.0271	0.0304	112.2%
coefficient of variation (%)	5.4	6.2	113.6%

**Table 8:** Experimental results of the loss factor η for Mat B.

Loss Factor	η_1_ (Before The Fatigue Test)	η_2_ (After The Fatigue Test)	η_2_/η_1_
mean value	0.4370	0.4294	98.3%
standard deviation	0.0208	0.0246	118.3%
coefficient of variation (%)	4.8	5.7	120.4%

## Data Availability

The data presented in this study are available on request from the corresponding author.
